# Overexpressed ITGA2 contributes to paclitaxel resistance by ovarian cancer cells through the activation of the AKT/FoxO1 pathway

**DOI:** 10.18632/aging.102954

**Published:** 2020-03-22

**Authors:** Linlin Ma, Yan Sun, Dan Li, Hansong Li, Xin Jin, Dianyun Ren

**Affiliations:** 1 Department of Obstetrics and Gynecology, Beijing Hospital, National Center of Gerontology, Institute of Geriatric Medicine, Chinese Academy of Medical Sciences, Beijing 100730, R.P. China; 2 Department of Pancreatic Surgery, Union Hospital, Tongji Medical College, Huazhong University of Science and Technology, Wuhan 430022, China; 3 Sino-German Laboratory of Personalized Medicine for Pancreatic Cancer, Union Hospital, Tongji Medical College, Huazhong University of Science and Technology, Wuhan 430022, China; 4 Cancer Center, Union Hospital, Tongji Medical College, Huazhong University of Science and Technology, Wuhan 430022, China; 5 Cardiovascular Medicine Department, Union Hospital, Tongji Medical College, Huazhong University of Science and Technology, Wuhan 430022, China

**Keywords:** integrin subunit α2 (ITGA2), PTX resistance, p-AKT, forkhead box O1 (FoxO1)

## Abstract

Ovarian cancer is one of the most malignant tumors of the female reproductive system, with high invasiveness. The disease is a severe threat to women's health. The ITGA2 gene, which codes for integrin subunit α2, is involved in the proliferation, invasion, and metastasis of cancer cells. Although previous studies have shown that ITGA2 increases in ovarian cancer, the specific molecular mechanism of how ITGA2 promotes ovarian cancer proliferation and metastasis is still unclear. In this study, we confirmed that ITGA2 was elevated in ovarian cancer, which led to poor prognosis and survival. Overexpressed ITGA2 promoted the proliferation of ovarian cancer cells. We also found that ITGA2 regulated the phosphorylation of forkhead box O1 (FoxO1) by mediating AKT phosphorylation, which provided a reasonable explanation for ITGA2’s role in ovarian cancer’s resistance to albumin paclitaxel. In summary, ITGA2 could be used as a new therapeutic target and prognostic indicator in ovarian cancer.

## INTRODUCTION

Ovarian cancer is one of the most malignant tumors of the female reproductive system; it is highly invasive and deadly [1]. There are six different histologic subtypes of ovarian cancer, including mucinous, serous, transitional-cell, endometroid, squamous carcinoma, and clear-cell cancers [2]. As the most common histological subtype, high-grade serous ovarian carcinoma (HGSOC) accounts for more than 70% of epithelial ovarian cancer (EOC) [3]. Because of the ovary’s deep position and small volume, most ovarian cancers enter the advanced stage, with a five-year survival rate of only 47% [4]. Thus, it is necessary to explore the specific molecular pathogenesis and find early diagnosis and treatment strategies for ovarian cancer.

As one of the important members in the family of adhesion molecules, integrins are critical to multiple biological functions, such as inflammation, inflammatory response, and thrombogenesis [5]. These adhesion molecule types belong to heterodimeric transmembrane proteins composed of alpha (α) and beta (β) components [6]. The alpha (α) subunit is closely related to the formation of the extracellular matrix (ECM), while the beta (β) subunit is essential for the regulation of intracellular signaling cascades (FAK, AKT, ROCK). Recent literature suggests that integrins can mediate cell-matrix and cell-cell interactions, which have been identified as significant in accelerating the incidence and development of tumors [7, 8]. There is also evidence that integrin subunit α2 promotes the invasion of peritoneal tumor aggregates or spheres [9].

The ITGA2 gene codes for integrin subunit α2 and is known to participate in the proliferation, invasion, and metastasis of cancer cells [9–11]. The ITGA2 gene is highly expressed in ovarian cancer, as established by transcriptomics and histochemistry studies of the ovary and single follicles [12]. However, although previous investigations have provided a foundation for our research to a certain degree, the specific molecular mechanism of how ITGA2 promotes ovarian cancer proliferation and metastasis is still unclear.

Given the potential therapeutic targets of ITGA2, we systematically explored the significance of ITGA2 overexpression in ovarian cancer. Firstly, we verified that the expression level of ITGA2 in ovarian cancer is indeed elevated. Then, we conducted cell biology function and animal experiments to reaffirm the promoting role of ITGA2 in the proliferation of ovarian cancer cells. Lastly, we sought to identify the mechanism by which ITGA2 enhances the spread of ovarian cancer cells.

## RESULTS

### Overexpressed ITGA2 correlated with poor prognosis in ovarian cancer

**Figure 1:**
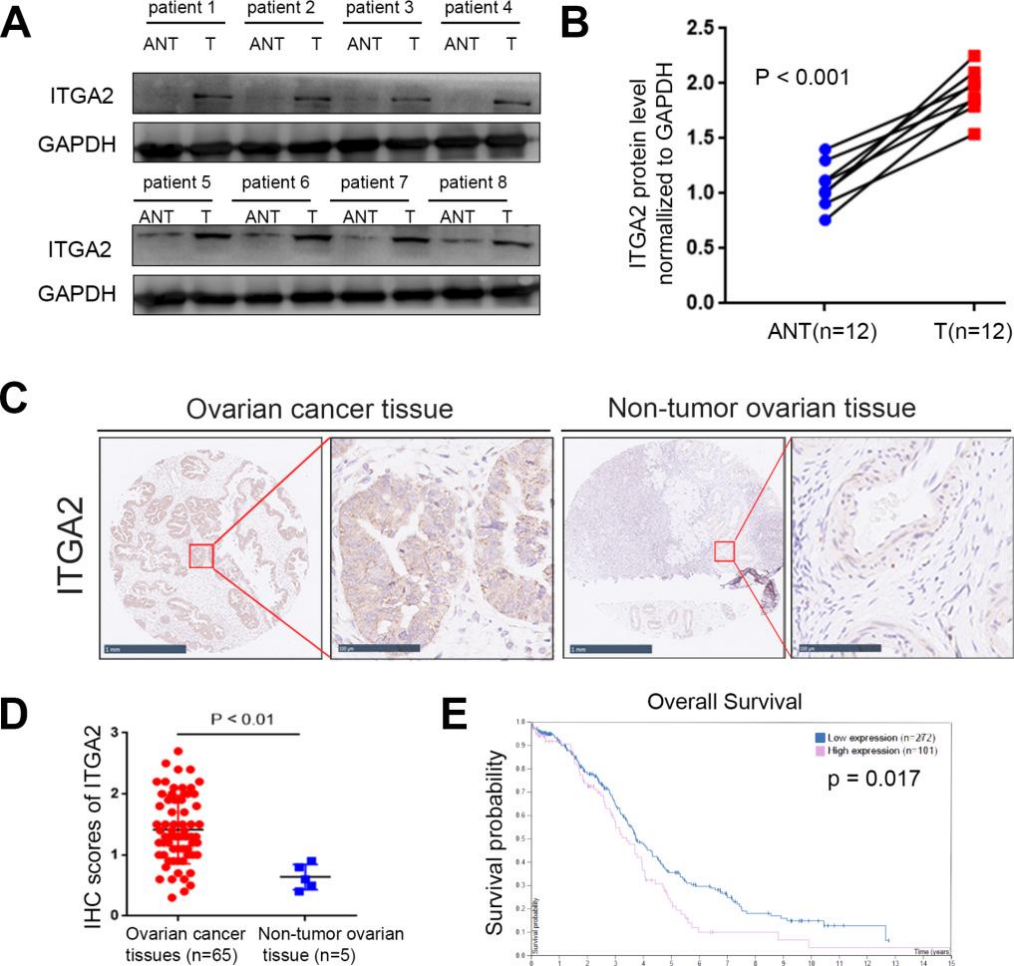
**The overexpressed ITGA2 is correlated with poor prognosis in ovarian cancer.** (**A**, **B**) Western blot assay was conducted to explore the protein expression of ITGA2 in 8 paired primary ovarian cancer tissues (T) and the matched adjacent normal tissues (ANT) of the same patient (**A**). The quantified proteins expression level of ITGA2 were also shown (**B**). The P values were shown in the figure. Statistical analyses were performed with D’Agostino and Pearson omnibus normality test. (**C**) TMA tissue sections were used for ITGA2 IHC staining. The IHC images were shown. The scale bars were shown in the figure. (**D**) Dot plots to show the IHC score of ITGA2 expression using TMA tissue sections (normal ovarian specimens: n = 5, ovarian cancer TMA specimens: n = 65, P < 0.001). Statistical analyses were performed with D’Agostino and Pearson omnibus normality test. (**E**) The overall survival of ovarian cancer patients was searched by Human Protein Atlas database (P < 0.001).

As mentioned in the introduction, ITGA2 is overexpressed in several malignant tumors [13, 14], but its expression level in ovarian cancer remains unclear. To determine ITGA2 expression levels in ovarian cancer, eight primary ovarian cancer samples with matching adjacent normal ovarian tissues were examined using Western Blot analysis. The results showed that the level of ITGA2 increased significantly in ovarian cancer (Figure 1A and 1B).

To determine the clinical relevance of ITGA2 in ovarian cancer, we assessed 70 paraffin-embedded, archived ovarian cancer tissues immunohistochemically (IHC) (Figure 1C and 1D). Consistent with our findings from the Western Blot analysis, we established that ITGA2 was markedly overexpressed in ovarian cancer (Figure 1C and 1D). Additionally, our study of the human protein atlas web tool revealed that ITGA2 correlated significantly with the unfavorable prognosis of ovarian cancer patients (Figure 1E). Collectively, these results suggest that ITGA2 might be a prognostic biomarker of ovarian cancer.

### Silencing ITGA2 inhibited the aggressiveness of ovarian cancer *in vitro*

**Figure 2:**
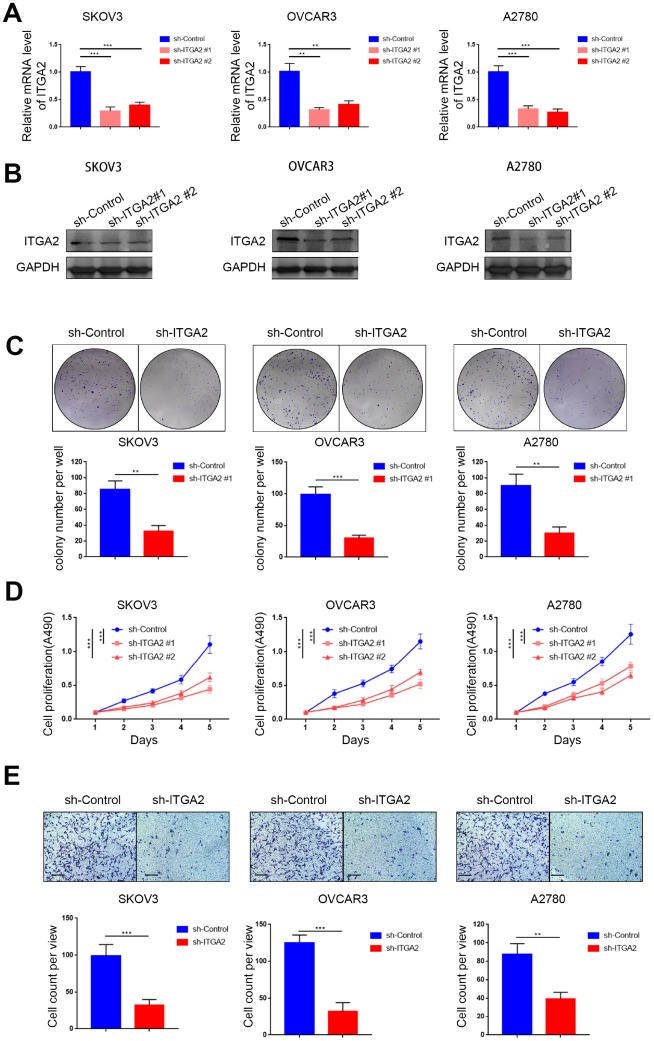
**Silencing ITGA2 inhibited the aggressiveness of ovarian cancer *in vitro.*** (**A**). SKOV3, OVCAR3, and A2780 cells lines with ITGA2 knockdown were established. RT-PCR assay (**A**) and Western blot assay (**B**) were performed in the three cell lines. GAPDH served as an internal reference. Data presented as the mean ± SD of three independent experiments. Each sh-ITGA2 group was compared with sh-Control group. Statistical analyses were performed with one-way ANOVA followed by Tukey's multiple comparison's tests. **, P < 0.01; ***, P < 0.001. c-e. SKOV3, OVCAR3, and A2780 cells lines with ITGA2 knockdown were established. The cells were harvested for colony formation assay (**C**), MTS assay (**D**), and migration assay (**E**). For e, representative images of migrated cells were shown based on a transwell assay. Each bar represents the mean ± SD of three to five independent experiments. Each sh-ITGA2 group was compared with sh-Control group. Statistical analyses were performed with one-way ANOVA followed by Tukey's multiple comparison's tests. **, P < 0.01; ***, P < 0.001.

To explore the biological role of ITGA2 in the progression of ovarian cancer, we knocked down ITGA2 in SKOV3, OVCAR3, and A2780 cell lines (Figure 2A, 2B). Colony formation and MTS assays indicated that knocking down ITGA2 significantly inhibited the proliferation ability of ovarian cancer (Figure 2C, 2D). Furthermore, our results showed that the invasion ability of ovarian cancer cells correlated positively with the expression level of ITGA2 (Figure 2E). Thus, our data indicate that silencing ITGA2 could inhibit the aggressiveness of ovarian cancer *in vitro*.

### Overexpressed ITGA2 promoted the aggressiveness of ovarian cancer *in vitro*

**Figure 3:**
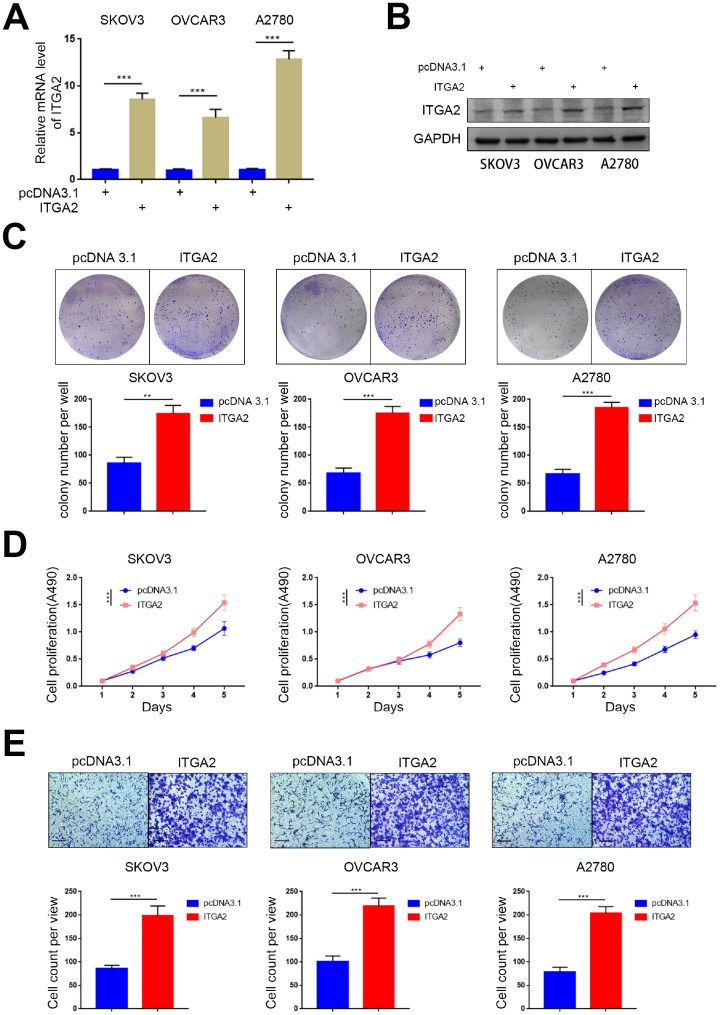
**Overexpressed ITGA2 promoted the aggressiveness of ovarian cancer *in vitro.*** (**A**) SKOV3, OVCAR3, and A2780 cells lines with stably overexpressed ITGA2 were established. RT-PCR assay (**A**) and Western blot assay (**B**) were performed in the three cell lines. GAPDH served as an internal reference. Data presented as the mean ± SD of three independent experiments. Each sh-ITGA2 group was compared with sh-Control group. Statistical analyses were performed with one-way ANOVA followed by Tukey's multiple comparison's tests. ***, P < 0.001. (**C**–**E**) SKOV3, OVCAR3, and A2780 cells lines with stably overexpressed ITGA2 were established. The cells were harvested for colony formation assay (**C**), MTS assay (**D**), and migration assay (**E**). For e, representative images of migrated cells were shown based on a transwell assay. Each bar represents the mean ± SD of three to five independent experiments. Each sh-ITGA2 group was compared with sh-Control group. Statistical analyses were performed with one-way ANOVA followed by Tukey's multiple comparison's tests. **, P < 0.01; ***, P < 0.001.

To explore the biological role of overexpressed ITGA2 in ovarian cancer progression even further, we established SKOV3, OVCAR3, and A2780 cell lines with stably overexpressed ITGA2 (Figure 3A, 3B). Colony formation and MTS assays revealed that the overexpression of ITGA2 significantly improved the proliferation ability of ovarian cancer (Figure 3C, 3D). Furthermore, the invasion transwell assay showed that the invasion ability of ovarian cancer cells was enhanced with the overexpression of ITGA2 (Figure 3E). Therefore, these results suggest that overexpressed ITGA2 could promote the aggressive nature of ovarian cancer cells *in vitro*.

### ITGA2 contributed to the activation of the AKT pathway in ovarian cancer cells

**Figure 4:**
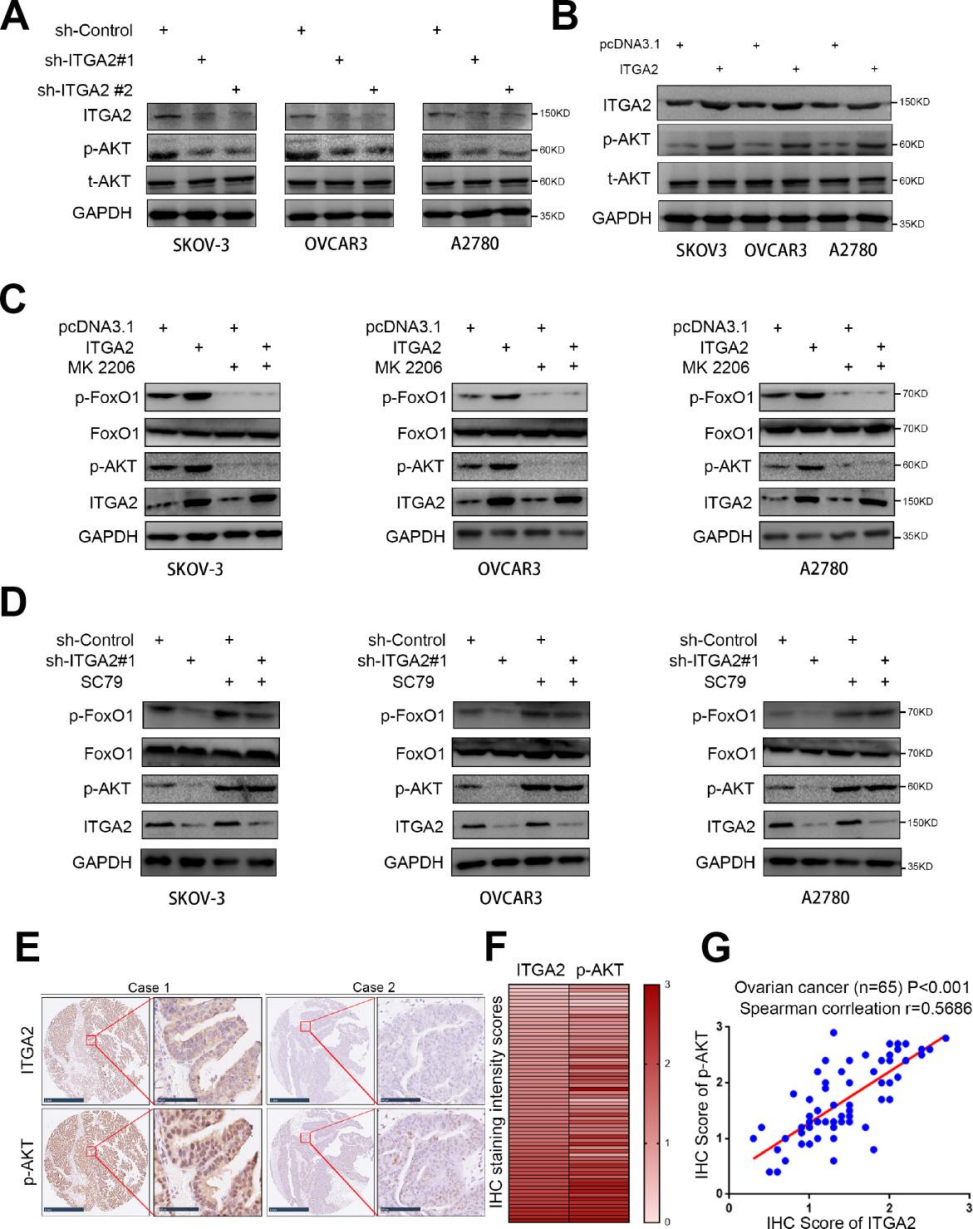
**ITGA2 contributes to activating AKT pathway in ovarian cancer cells.** (**A**) SKOV3, OVCAR3, and A2780 cells lines were infected with sh-Control, sh-ITGA2 #1, or sh-ITGA2 #2. Western blot analysis was performed with these cells after 72 hours culturing. GAPDH served as an internal reference. (**B**) SKOV3, OVCAR3, and A2780 cells lines were infected with or without ITGA2 plasmids. Western blot analysis was performed with these cells after 72 hours culturing. GAPDH served as an internal reference. (**C**) SKOV3, OVCAR3, and A2780 cells lines were infected with or without ITGA2 plasmids and were treated with or without AKT inhibitor (MK 2206). Western blot analysis was performed with these cells after 72 hours culturing. GAPDH served as an internal reference. (**D**) SKOV3, OVCAR3, and A2780 cells lines were infected with sh-Control, sh-ITGA2 #1, or sh-ITGA2 #2 and were treated with or without AKT agonist (SC79). Western blot analysis was performed with these cells after 72 hours culturing. GAPDH served as an internal reference. (**E**) IHC Images of ITGA2 and p-AKT staining using TMA tissue sections (n = 65 ovarian cancer patient specimens). The scale bars were shown as indicated. f and g. Heatmap (**F**) and dot plot (**G**) to show the correlation of IHC scores for the expression of the ITGA2 and p-AKT proteins in ovarian cancer patient specimens. (r = 0.5686 for spearman correlation coefficients, P < 0.001).

The AKT pathway is frequently activated in ovarian cancer and contributes to its progression [15]. Therefore, we questioned whether ITGA2 might regulate the aggressive ability of ovarian cancer cells by activating the AKT pathway. As shown in Figure 4A, 4B, silencing ITGA2 inhibited the phosphorylation of AKT, while overexpressing ITGA2 increased it. But both silencing and overexpressing ITGA2 had no impact on the expression level of total AKT. Additionally, an AKT inhibitor (MK 2206) reversed the improved phosphorylation level of AKT by overexpressing ITGA2 (Figure 4C), while an AKT agonist (SC79) restored the inhibited phosphorylation level of AKT by knocking down ITGA2 (Figure 4D). Furthermore, as a direct downstream target of the AKT pathway [16], the phosphorylation level of FOXO1 changed with the inhibition or activation of the AKT pathway, regulated by ITGA2 expression level (Figure 4C, 4D).

To determine the clinical relationship between ITGA2 and p-AKT in human ovarian cancer specimens, we analyzed the protein expression level of ITGA2 and phosphorylation level of AKT in a cohort of ovarian cancer patients immunohistochemically (n = 65). The results showed that there was a positive correlation between the protein levels of ITGA2 and the phosphorylation levels of AKT in ovarian cancer patients (Figure 4E–4G). So, ITGA2 is potentially responsible for activating AKT signaling in ovarian cancer.

### ITGA2 knockdown overcame paclitaxel resistance in ovarian cancer *in vitro*

**Figure 5:**
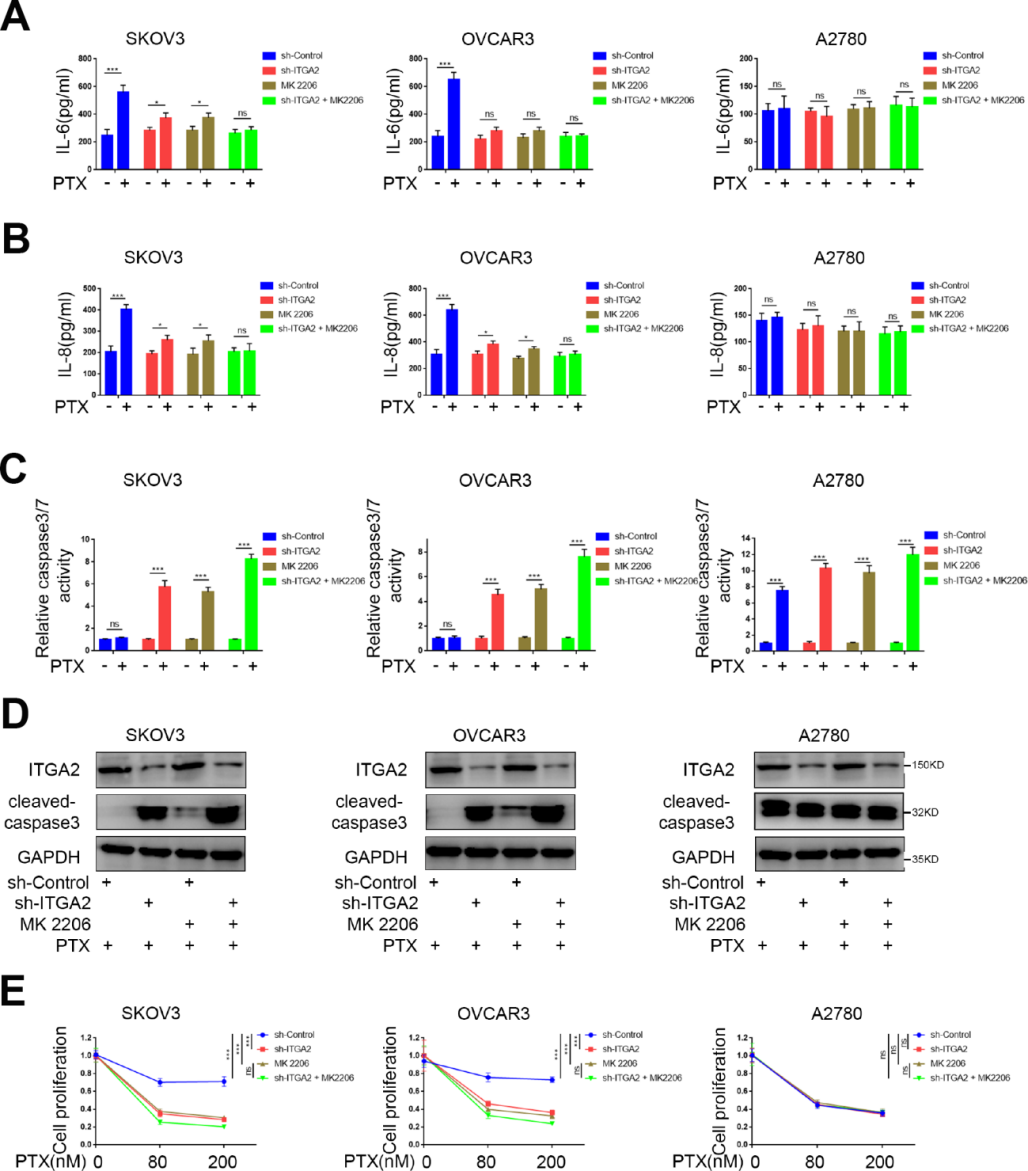
**ITGA2 knockdown overcame drug-resistance induced by paclitaxel in ovarian cancers *in vitro*.** (**A**–**D**) SKOV3, OVCAR3, and A2780 cells lines were infected sh-Control or sh-ITGA2. Tumor cells were cultured at a density of 5x10^5^ cells/well with or without the treatment of MK 2206 and with or without the treatment of PTX. After treatment for forty-eight hours, the tumor cells supernatants were collected to tested for levels of IL-6 (**A**) and IL-8 (**B**) and the tumor cells were harvested for caspase-Glo 3/7 assay (**C**) and western blot assay (**D**). Each bar represents the mean ± SD of three independent experiments. PTX treatment group was compared with no PTX treatment group. Statistical analyses were performed with one-way ANOVA followed by Tukey's multiple comparison's tests. ns, no significant; *, P < 0.05; ***, P < 0.001. (**E**) SKOV3, OVCAR3, and A2780 cells lines were infected sh-Control or sh-ITGA2. 48 hours post-infection, the cells were harvested for MTS assay with the treatment of PTX. Each bar represents the mean ± SD of five independent experiments. Each group was compared with sh-Control group. Statistical analyses were performed with one-way ANOVA followed by Tukey's multiple comparison's tests. **, P < 0.01; ***, P < 0.001.

Since FOXO1 is linked to cytotoxic stress induced by paclitaxel and contributes to drug-resistance in ovarian cancers [17], we supposed that ITGA2 might play an important biological role in drug-resistance in ovarian tumors. SKOV3 and OVCAR3 cell lines are reportedly PTX-resistant, while the A2780 cell line is PTX-sensitive [18]. Consistently, the two potential mediators of PTX resistance, IL-6 and IL-8 [19, 20] increased significantly in SKOV3 and OVCAR3 cell lines but not in the A2780 cell line (Figure 5A, 5B). Additionally, ITGA2 knockdown or AKT inhibition substantially decreased the production of IL-6 and IL-8 induced by PTX treatment, and combining ITGA2 knockdown with AKT inhibition synergistically reduced the production of IL-6 and IL-8 caused by PTX treatment (Figure 5A, 5B).

Furthermore, we evaluated the relative caspase-3/7 activity and the expression level of caspase-3 and found that ITGA2 knockdown or AKT inhibition significantly increased cell apoptosis induced by PTX (Figure 5C, 5D). Consistent with this finding, the MTS assay confirmed that ITGA2 knockdown or AKT inhibition significantly decreased cell proliferation after treatment of SKOV3 and OVCAR3 cell lines with PTX (Figure 5E and Supplementary Figure 1). These results suggest that ITGA2 knockdown or/and AKT inhibition could inhibit PTX resistance in PTX-resistant ovarian cancer cell lines separately and synergistically.

### ITGA2 knockdown overcame paclitaxel resistance in ovarian cancer *in vivo*

**Figure 6:**
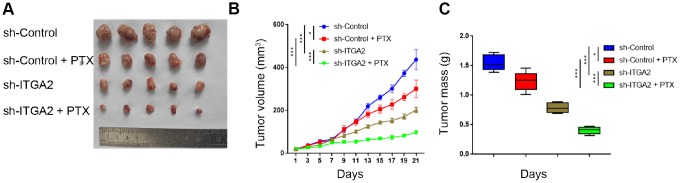
**ITGA2 knockdown overcame drug-resistance induced by paclitaxel in ovarian cancers *in vivo*.** (**A**–**C**) After 72 hours postinfection, SKOV3 cells infected with sh-Control or sh-ITGA2 were subcutaneously injected into nude mice. Mice with subcutaneous SKOV3 tumors (n = 5/group) were treated with or without PTX (7.5 mg/ kg) for five consecutive days per week were harvested and photographed (a) on day 21. Data on tumor volume (**B**) and tumor mass (**C**) were shown as means ± SD (n = 5). sh-Control group was compared with sh-ITGA2 group or PTX group, sh-ITGA2 group was compared with sh-ITGA2+PTX group. Statistical analyses were performed with two-way ANOVA followed by Sidak's multiple comparison's tests. ns, not significant; ***, P < 0.001.

We used the SKOV3 cell line to study the biological role of ITGA2 in PTX resistance in ovarian cancer *in vivo*. As Figure 6A–6C shows, PTX treatment alone had a limited impact on the inhibition of tumor growth caused by PTX resistance in cell lines. Tumors formed by ITGA2-silenced SKOV3 cells were smaller and lighter than those created by ITGA2-normally expressed cells. Combining ITGA2 knockdown with PTX treatment caused a more remarkable decrease in tumor volume and tumor mass, suggesting that knocking down ITGA2 could inhibit PTX resistance and also enhance the therapeutic ability of PTX in ovarian cancer *in vivo*.

## DISCUSSION

ITGA2 has been reported by previous studies to promote tumor progression, both *in vivo* and *in vitro*, in many tumors [21–23], therefore, playing an essential role in cell migration, invasion, survival, and angiogenesis [24, 25]. Also, the expression level of ITGA2 increases in pancreatic cancer due to promoter hypomethylation [26]. In this study, we reveal that the ITGA2 protein increased in ovarian cancer tissues compared to normal ovarian tissues, and ITGA2 correlated significantly with the unfavorable prognosis of ovarian cancer, suggesting that ITGA2 could be regarded as a prognostic marker of ovarian cancer. We also demonstrated the tumor-promoting effect of ITGA2 in ovarian cancer *in vitro* and *in vivo*.

More and more evidence show that ITGA2 acts as a cancer-promoting protein to enhance tumor proliferation and metastasis [7, 8, 21], although there is still some evidence to the contrary in breast [27] and colon cancers [28]. Our results here demonstrate that the increased expression of ITGA2 in ovarian cancer promoted the malignant biological behavior of tumor cells, which is consistent with findings in studies on pancreatic cancer [29], liver cancer [30], Hodgkin lymphoma [31], and osteosarcoma [32].

ITGA2 might be involved in tumor transformation or development, and it enriches several signaling pathways related to cell adhesion and migration [33], but its role in the PI3K/AKT signaling pathway has not been established. The PI3K/AKT pathway is usually activated abnormally in most tumors, enabling the levels of phosphorylated AKT to increase, which is vital to cell growth, proliferation, differentiation, movement, survival, and metabolism [34, 35]. In this investigation, we showed that ITGA2 promoted AKT phosphorylation and further accelerated the phosphorylation of the oncogenic protein FOXO1. The FOXO protein family belongs to a class of transcription factors that includes FoxO1, FoxO3a, and FoxO4. The activity of FOXO1 can be regulated by phosphorylation or dephosphorylation as a downstream molecule of the PI3K/AKT signaling pathway [36]. FoxO1 is a significant regulator of redox balance, whose changes could stimulate cell proliferation and differentiation and inhibit cell apoptosis [37].

Interestingly, our research suggests that ITGA2 could promote the phosphorylation of AKT in ovarian cancer, thereby enhancing the proliferation of ovarian cancer cells. The phosphorylation level of FOXO1 also decreased or increased when we respectively knocked down or overexpressed ITGA2. Moreover, when we used AKT agonists and inhibitors to treat cells separately, we found that phosphorylated FoxO1 was enhanced or weakened with the enhancement or decrease of phosphorylated AKT, respectively, no matter how ITGA2 changed, which indicates that ITGA2 regulated the biological behavior of ovarian cancer cells by acting on the AKT/FOXO1 axis. Our research results provide a reasonable explanation for ITGA2’s stimulation of the proliferation of ovarian cancer cells.

Albumin paclitaxel has been used extensively in oncotherapy. However, the therapeutic effect of this drug on tumors, such as urothelial cancer [38] and pancreatic cancer [39], is poor because of drug resistance. As first-line chemotherapy for ovarian cancer, tumor cells have been found to develop resistance to albumin paclitaxel [40], but the specific mechanism of how this unfolded remained unclear. Encouragingly, our results showed that the sensitivity of ovarian cancer cells to albumin paclitaxel improved significantly after we knocked down ITGA2 expression. ITGA2 might, hence, become a therapeutic target. Our findings suggest that knocking down ITGA2 could significantly reduce PTX resistance by ovarian cancer. The means of decreasing ITGA2 expression potentially has high value for clinical applications. Xu Y et al revealed that BMSCs-derived exosomes overexpressing miR-16-5p repressed colorectal cancer (CRC) growth by decreasing ITGA2 levels [41]. We, therefore, have drawn up a further study to assess the impact of combining PTX treatment with BMSCs-derived exosomes overexpressing miR-16-5p.

There were some limitations to our research. Firstly, we did not investigate the reason for the increase in ITGA2 expression in ovarian cancer. One study found that the level of ITGA2 promoter methylation in pancreatic cancer reduced, resulting in elevated ITGA2 expression [26], but we cannot vouch for that observation here. The mechanism of how ITGA2 is up-regulated in ovarian cancer, therefore, requires further research. Secondly, we did not explore the mechanism of how ITGA2 regulated the AKT/Foxo1 axis.

Collectively, our study suggests that aberrant expression of ITGA2 increases ovarian cancer cell proliferation and enhances ovarian cancer cell resistance to albumin paclitaxel through the AKT/FOXO1 signaling axis.

## MATERIALS AND METHODS

### Cell lines and cell culture

Ovarian cancer cell lines (SKOV3, OVCAR3, and A2780) were purchased from the Chinese Academy of Science Cell Bank and cultured in an RPMI 1640 medium containing 10% fetal bovine serum (FBS) (HyClone, USA), 100 μg/ml streptomycin, and 100 U/ml penicillin at 37°C and 5% CO_2_.

### Patients and tissue specimens

We collected ovarian cancer tissues and their matching adjacent tissues from 30 patients admitted to the Department of Obstetrics and Gynecology, Wuhan Union Hospital from July 2018 to July 2019. Part of the fresh specimens was stored in liquid nitrogen, and the other part was fixed in a formaldehyde fixative. All patients signed written informed consent before providing samples. Our local ethics committee (Tongji Medical College, HUST, China) approved our research.

### Plasmids, reagent, and antibodies

Flag-ITGA2 plasmids were constructed by Shanghai Genechem Co., LTD. Human expression vectors for flag-ITGA2 recombinant proteins were generated using the pcDNA3.1 backbone vector, as described previously [42]. Selected antibodies included ITGA2 (Abcam, ab133557, dilution ratio 1:1000), GAPDH (Proteintech, 10494-1-AP, dilution ratio 1:2000), AKT (Cell Signaling Technology, 2920, dilution ratio 1:1000), pAKT-S473 (Cell Signaling Technology, 4060, dilution ratio 1:1000), TLR4 (Proteintech, 19811-1-AP, dilution ratio 1:1000), and Caspase-3 (Proteintech, 19677-1-AP, dilution ratio 1:1000). The reagent used was Paclitaxel (MedChemExpress, HY-B0015, working concentration 80 nM *in vitro* and 10 mg/kg *in vivo*).

### Tissue microarray (TMA) and immunohistochemistry (IHC)

Ovarian cancer TMA slides were purchased from Outdo Biobank (Shanghai, China). The clinical characteristics of the analyzed TMA patients are provided in Supplementary Table 3. An ITGA2 antibody (Abcam, ab133557, dilution ratio 1:500) was used for the immunohistochemical staining of the slides according to standard IHC, and, additionally, a Caspase-3 antibody (Proteintech, 19677-1-AP, dilution ratio 1:1000) was used for the immunohistochemical staining of nude mice xenografts. TMA slides were then observed and immunohistochemistry was scored using an optical microscope. Two experienced pathologists who took no part in the experiments evaluated the immune score independently. We used the product of the staining intensity score and the ratio of positive tumor cells as the staining index (SI). The staining intensity scoring criteria were defined as follows: 1 = weak staining at 100× magnification but little or no staining at 40× magnification; 2 = medium staining at 40× magnification; 3 = strong staining at 40× magnification.

### Lentivirus and transduction

We purchased ITGA2-overexpressed lentiviruses, ITGA2-knocked-down lentiviruses, and corresponding negative control lentiviruses from GenePharma (Shanghai, China). All lentiviral vectors expressed enhanced green fluorescent protein (GFP) and puromycin resistant genes. We used a Lipofectamine 2000 reagent (Invitrogen) to transduce ITGA2-overexpressed and ITGA2-knocked-down lentiviral systems into 293T cells. The transfection medium was replaced with DMEM containing 10% FBS and 1 mM sodium pyruvate 24 hours after transfection. We added a viral culture to ovarian cancer cell lines with 12 μg/ml of polybrene after 48 hours of incubation. After 48 hours of transduction, we used 10 μg/ml of puromycin to screen for virus-infected cells. Specific sequence information for shRNA is provided in Supplementary Table 1.

### Western blot analysis

We lysed harvested cells using a RIPA lysate (Beyotime, China) containing protease inhibitors to extract total protein. A BCA protein concentration determination kit (Cwbiotech, China) was used to determine protein concentration. The obtained cell lysates were centrifuged at 12000 rpm for 10 minutes at 4°C after ultrasonic cracking. We then transferred the resulting supernatant to a clean 1.5 ml centrifuge tube and mixed quarter volume protein loading buffer. The product samples were boiled for 10 minutes, separated using SDS-PAGE for further analysis, transferred to nitrocellulose membranes, blocked with 5% skim milk powder for 1 hour, and then incubated overnight with specific antibodies at 4°C. The following day, the membranes were washed three times with TBST (10 minutes each time), incubated with a horseradish peroxidase-based secondary antibody for 1 hour at room temperature, and their proteins were detected using an ECL illuminating liquid under the illumination of X-rays.

### Real-time PCR

We used a Trizol reagent (Thermo Fisher Scientific, USA) to extract total RNA from cells and determined the extracted RNA concentration with a NanoDrop 2000 (Thermo, USA). Following standard protocol (PrimeScript™ RT reagent Kit), RNA samples were subjected to reverse transcription to synthesize stable cDNA, which was then amplified using a PCR kit (TB Green™ Fast qPCR Mix) for further analysis. GAPDH gene expression was used as a control. The relative expression levels of mRNA were determined using the 2^-ΔCq^ method. Supplementary Table 2 contains the forward and reverse primer sequences of ITGA2, TLR4, and GAPDH.

### Transwell invasion assay

Ovarian cancer cells resuspended in serum-free RPMI 1640 were inoculated into the upper transwell chamber containing Matrigel, while RPMI 1640 containing 15% fetal bovine serum was added to the lower chamber as a chemical inducer. After 12-24 hours of incubation at 37°C and 5% CO_2_, the chambers were stained with prepared crystal violet, and the number of migrated cells was observed under a light microscope and photographed with computers.

### Cell proliferation and clone formation assay

We used the MTS (3-(4,5-dimethylthiazol-2-yl)-5-(3-carboxymethoxyphenyl)-2-(4-sulfophenyl)-2H-tetrazolium, inner salt) assay to assess cell viability. 2000 cells from the control group, ITGA2-knockdown group, and ITGA2-overexpression group were inoculated into each well of 96-well plates. Albumin paclitaxel (PTX) was then added to the plates according to a preset concentration gradient. To each well was also added 20 μl of MTS after continuous cultivation for 72 hours. We measured the absorbance of the wells at a wavelength of 490 nm using a microplate reader and calculated the IC50 of each group at the end.

We used 6-well plates for plate colony formation experiments. 500 cells were counted from all treatment groups and seeded into the 6-well plates. The medium in each plate was changed every three days. Cells were fixed with 4% paraformaldehyde for 20 minutes after 12-14 days of culture, and the cell colonies were stained with prepared purple crystal for 30 minutes.

### Caspase-Glo 3/7 assay [43]

We mixed a protein solution with an equilibrated Caspase-Glo 3/7 reagent (Promega Corporation, Madison, USA) in a 1:1 proportion, *i.e.*, 50 μL of the total volume contained 10 μg of protein and 50 μL of the Caspase-Glo 3/7 reagent. The product, a miscible liquid, was incubated for 1 hour at an indoor temperature, and its luminescence was measured using a TD 20/20 Luminometer (Turner Designs, Sunnyvale, CA, USA). We repeated each procedure three times to ensure the accuracy of the experimental results.

### Tumor xenograft model

We purchased BALB/c nude mice (5 weeks of age, 18-20g) from Vitalriver (Beijing, China) and used them for xenotransplantation. The nude mice were divided into 4 different groups, including shControl, shITGA2, shControl+Albumin paclitaxel, and shITGA2+Albumin paclitaxel. Each group contained 6 nude mice. 5 × 10^6^ cells were injected subcutaneously into the right-back of each mouse. The length and width of xenografts were measured with a vernier caliper every three days, and the tumor volumes were calculated using the equation (L×W^2^)/2. Mice were euthanized and dissected to collect xenografts 21 days after injecting tumor cells. Our local ethics committee (Tongji Medical College, HUST, China) approved all experimental animal procedures.

### Statistical analysis

Data were expressed as mean ± SD of at least three independent and repetitive experiments. Statistical significance was assessed using the paired t-test, Student's t-test, and one or two-way ANOVA, followed by Tukey's multiple comparison tests. *P*<0.05 was considered statistically significant.

## Supplementary Material

Supplementary Figure 1

Supplementary Tables 1 and 2

Supplementary Table 3
