# Correction for: Overexpressed ITGA2 contributes to paclitaxel resistance by ovarian cancer cells through the activation of the AKT/FoxO1 pathway

**DOI:** 10.18632/aging.203703

**Published:** 2021-11-30

**Authors:** Linlin Ma, Yan Sun, Dan Li, Hansong Li, Xin Jin, Dianyun Ren

**Affiliations:** 1 Department of Obstetrics and Gynecology, Beijing Hospital, National Center of Gerontology, Institute of Geriatric Medicine, Chinese Academy of Medical Sciences, Beijing 100730, R.P., China; 2 Department of Pancreatic Surgery, Union Hospital, Tongji Medical College, Huazhong University of Science and Technology, Wuhan, 430022, China; 3 Sino-German Laboratory of Personalized Medicine for Pancreatic Cancer, Union Hospital, Tongji Medical College, Huazhong University of Science and Technology, Wuhan, 430022, China; 4 Cancer Center, Union Hospital, Tongji Medical College, Huazhong University of Science and Technology, Wuhan, 430022, China; 5 Cardiovascular Medicine Department, Union Hospital, Tongji Medical College, Huazhong University of Science and Technology, Wuhan, 430022, China

**Keywords:** correction

Original article: Aging. 2020; 12:5336–5351.  . https://doi.org/10.18632/aging.102954

**This article has been corrected:** The authors asked to replace the section “**Patients and tissue specimens**” of Material and Methods due to the mistake in the source of the ovarian cancer tissues.

**Old text:**

We collected ovarian cancer tissues and their matching adjacent tissues from 30 patients admitted to the Department of Obstetrics and Gynecology, Wuhan Union Hospital from July 2018 to July 2019. Part of the fresh specimens was stored in liquid nitrogen, and the other part was fixed in a formaldehyde fixative. All patients signed written informed consent before providing samples. Our local ethics committee (Tongji Medical College, HUST, China) approved our research.

**New Text:**

We collected ovarian cancer tissues and their matching adjacent tissues from 10 patients admitted to the Department of Obstetrics and Gynecology, Beijing Hospital from July 2018 to July 2019. Part of the fresh specimens was stored in liquid nitrogen, and the other part was fixed in a formaldehyde fixative. All patients signed written informed consent before providing samples. The local ethics committee of Beijing Hospital approved this research.

