# Supplementary material for: Overexpressed ITGA2 contributes to paclitaxel resistance by ovarian cancer cells through the activation of the AKT/FoxO1 pathway
Source: Aging (Albany NY). 2020 Mar 22;12(6):5336–51. doi: 10.18632/aging.102954 (PMC7138566; doi:10.18632/aging.102954)
Supplement: Supplementary Figure 1 [file aging-12-102954-s002..pdf]

SUPPLEMENTARY FIGURE

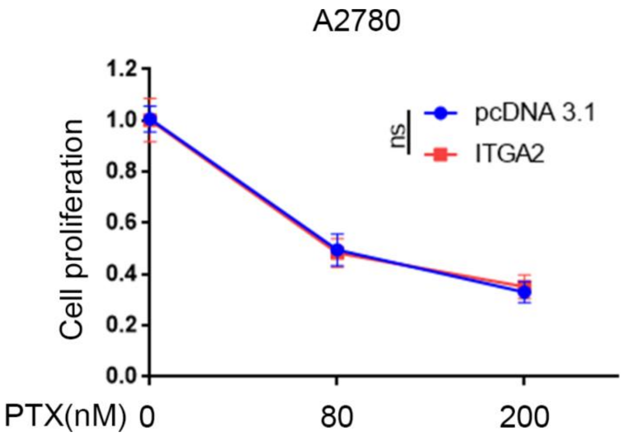

Supplementary Figure 1. A2780 cells lines were infected pcDNA 3.1 or ITGA2. 48 hours post-infection, the cells were harvested for MTS assay with the treatment of PTX. Each bar represents the mean ± SD of five independent experiments. pcDNA 3.1 group was compared with ITGA2 group. Statistical analyses were performed with one-way ANOVA followed by Tukey's multiple comparison's tests. ns, not significant.
