# Supplementary material for: Overexpressed ITGA2 contributes to paclitaxel resistance by ovarian cancer cells through the activation of the AKT/FoxO1 pathway
Source: Aging (Albany NY). 2020 Mar 22;12(6):5336–51. doi: 10.18632/aging.102954 (PMC7138566; doi:10.18632/aging.102954)
Supplement: Supplementary Tables 1 and 2 [file aging-12-102954-s003..pdf]

SUPPLEMENTARY TABLES

Supplementary Table 1. Sequences of RT-qPCR primers.

Species	Gene	Forward (5'-3')	Reverse (5'-3')
Human	GAPDH	ACCCAGAAGACTGTGGATGG	TTCAGCTCAGGGATGACCTT
Human	ITGA2	GGGAATCAGTATTACACAACGGG	CCACAACATCTATGAGGGAAGGG

Supplementary Table 2. Sequences of gene-specific shRNAs and siRNAs.

si-Control	purchased from RIBOBIO
sh-ITGA2 #1	CCGGCCGGCCAGATAGTGCTATATACTCGAGTATATAGCACTATCTGGCCGGTTTTTG
sh-ITGA2 #2	CCGGATGGCAATATCACGGTTATTCCTCGAGGAATAACCGTGATATTGCCATTTTTTG
