# Supplementary material for: Overexpressed ITGA2 contributes to paclitaxel resistance by ovarian cancer cells through the activation of the AKT/FoxO1 pathway
Source: Aging (Albany NY). 2020 Mar 22;12(6):5336–51. doi: 10.18632/aging.102954 (PMC7138566; doi:10.18632/aging.102954)
Supplement: Supplementary Table 3 [file aging-12-102954-s001..docx]

**Supplementary Table 3. The clinical characteristics of patients contained in the TMA.**

| No. | Type | Age | Metastasis | Primary organ | Pathological type | Pathological stage |
| --- | --- | --- | --- | --- | --- | --- |
| 1 | cancer | 49 | No | Yes | Ovarian serous adenocarcinoma | Ⅰ |
| 2 | cancer | 52 | No | Yes | Ovarian serous adenocarcinoma | Ⅱ |
| 3 | cancer | 78 | No | Yes | Ovarian serous adenocarcinoma | Ⅱ |
| 4 | cancer | 49 | No | Yes | Ovarian serous adenocarcinoma | Ⅱ-Ⅲ |
| 5 | cancer | 69 | No | Yes | Ovarian serous adenocarcinoma | Ⅲ |
| 6 | cancer | 70 | No | Yes | Ovarian serous adenocarcinoma | Ⅰ |
| 7 | cancer | 54 | No | Yes | Ovarian serous adenocarcinoma | Ⅱ |
| 8 | cancer | 30 | No | Yes | Ovarian serous adenocarcinoma | Ⅱ |
| 9 | cancer | 68 | No | Yes | Ovarian serous adenocarcinoma | Ⅱ |
| 10 | cancer | 68 | No | Yes | Ovarian serous adenocarcinoma | Ⅱ |
| 11 | cancer | 42 | No | Yes | Ovarian serous adenocarcinoma | Ⅱ |
| 12 | cancer | 63 | No | Yes | Ovarian serous adenocarcinoma | Ⅱ |
| 13 | cancer | 50 | No | Yes | Ovarian serous adenocarcinoma | Ⅱ |
| 14 | cancer | 61 | No | Yes | Ovarian serous adenocarcinoma | Ⅱ |
| 15 | cancer | 64 | No | Yes | Ovarian serous adenocarcinoma | Ⅱ |
| 16 | cancer | 52 | No | Yes | Ovarian serous adenocarcinoma | Ⅱ |
| 17 | cancer | 53 | No | Yes | Ovarian serous adenocarcinoma | Ⅱ |
| 18 | cancer | 51 | No | Yes | Ovarian serous adenocarcinoma | Ⅱ |
| 19 | cancer | 36 | No | Yes | Ovarian serous adenocarcinoma | Ⅱ |
| 20 | cancer | 54 | No | Yes | Ovarian serous adenocarcinoma | Ⅱ |
| 21 | cancer | 60 | No | Yes | Ovarian serous adenocarcinoma | Ⅱ |
| 22 | cancer | 48 | No | Yes | Ovarian serous adenocarcinoma | Ⅱ-Ⅲ |
| 23 | cancer | 64 | No | Yes | Ovarian serous adenocarcinoma | Ⅱ-Ⅲ |
| 24 | cancer | 53 | No | Yes | Ovarian serous adenocarcinoma | Ⅱ-Ⅲ |
| 25 | cancer | 55 | No | Yes | Ovarian serous adenocarcinoma | Ⅱ-Ⅲ |
| 26 | cancer | 64 | No | Yes | Ovarian serous adenocarcinoma | Ⅱ-Ⅲ |
| 27 | cancer | 53 | No | Yes | Ovarian serous adenocarcinoma | Ⅱ-Ⅲ |
| 28 | cancer | 59 | No | Yes | Ovarian serous adenocarcinoma | Ⅱ-Ⅲ |
| 29 | cancer | 42 | No | Yes | Ovarian serous adenocarcinoma | Ⅱ-Ⅲ |
| 30 | cancer | 43 | No | Yes | Ovarian serous adenocarcinoma | Ⅱ-Ⅲ |
| 31 | cancer | 53 | No | Yes | Ovarian serous adenocarcinoma | Ⅱ-Ⅲ |
| 32 | cancer | 68 | No | Yes | Ovarian serous adenocarcinoma | Ⅲ |
| 33 | cancer | 64 | No | Yes | Ovarian serous adenocarcinoma | Ⅲ |
| 34 | cancer | 48 | No | Yes | Ovarian serous adenocarcinoma | Ⅲ |
| 35 | cancer | 49 | No | Yes | Ovarian serous adenocarcinoma | Ⅲ |
| 36 | cancer | 54 | No | Yes | Ovarian serous adenocarcinoma | Ⅲ |
| 37 | cancer | 46 | No | Yes | Ovarian serous adenocarcinoma | Ⅲ |
| 38 | cancer | 72 | No | Yes | Ovarian serous adenocarcinoma |  |
| 39 | cancer | 17 | No | Yes | Ovarian mucinous adenocarcinoma | Ⅰ |
| 40 | cancer | 61 | No | Yes | Ovarian mucinous adenocarcinoma | Ⅰ-Ⅱ |
| 41 | cancer | 53 | No | Yes | Ovarian mucinous adenocarcinoma | Ⅱ |
| 42 | cancer | 49 | No | Yes | Ovarian mucinous adenocarcinoma | Ⅱ |
| 43 | cancer | 73 | No | Yes | Ovarian mucinous adenocarcinoma | Ⅱ |
| 44 | cancer | 40 | No | Yes | Ovarian mucinous adenocarcinoma | Ⅱ |
| 45 | cancer | 58 | No | Yes | Ovarian mucinous adenocarcinoma | Ⅱ |
| 46 | cancer | 73 | No | Yes | Ovarian mucinous adenocarcinoma |  |
| 47 | cancer | 23 | No | Yes | Ovarian endometrioid adenocarcinoma | Ⅰ |
| 48 | cancer | 48 | No | Yes | Ovarian endometrioid adenocarcinoma | Ⅰ-Ⅱ |
| 49 | cancer | 55 | No | Yes | Ovarian endometrioid adenocarcinoma | Ⅱ |
| 50 | cancer | 36 | No | Yes | Ovarian endometrioid adenocarcinoma | Ⅱ |
| 51 | cancer | —— | No | Yes | Ovarian clear cell carcinoma | Ⅰ-Ⅱ |
| 52 | cancer | 52 | No | Yes | Ovarian clear cell carcinoma | Ⅱ |
| 53 | cancer | 44 | No | Yes | Ovarian clear cell carcinoma | Ⅱ |
| 54 | cancer | 54 | No | Yes | Ovarian clear cell carcinoma | Ⅱ |
| 55 | cancer | 86 | No | Yes | Ovarian transitional cell carcinoma | Ⅱ-Ⅲ |
| 56 | cancer | 58 | No | Yes | Mixed ovarian adenocarcinoma | Ⅱ |
| 57 | cancer | 38 | No | Yes | Mixed ovarian adenocarcinoma | Ⅰ-Ⅱ |
| 58 | cancer | 58 | No | Yes | Ovarian cancer sarcoma |  |
| 59 | cancer | 23 | No | Yes | Ovarian germ cell tumor |  |
| 60 | cancer | 20 | No | Yes | Ovarian germ cell tumor |  |
| 61 | cancer | 28 | No | Yes | Ovarian germ cell tumor |  |
| 62 | cancer | 37 | No | Yes | Ovarian germ cell tumor |  |
| 63 | cancer | 50 | No | Yes | Ovarian stromal cell tumor |  |
| 64 | cancer | —— | No | Yes | Ovarian stromal cell tumor |  |
| 65 | cancer | 71 | No | Yes | Ovarian stromal cell tumor |  |
